# Repurposing renin‐angiotensin system‐targeting medications for treatment of cerebral amyloid angiopathy and associated cognitive‐behavioral deficits in Tg‐SwDI mice

**DOI:** 10.1002/alz.095709

**Published:** 2025-01-09

**Authors:** Ariana Hernandez, Natalia Noto, Chana Vogel, Iliana Uribe, Julianna Bonetti, Eleanor Wind, Victoria Pulido‐Correa, Mikayla Jeneske, Bianca Echeverria, Yazmin Restrepo, Shay Moen, Vedika Chiduruppa, Robert Speth, Lisa S Robison

**Affiliations:** ^1^ Nova Southeastern University, Fort Lauderdale, FL USA; ^2^ Miami Dade College, Miami, FL USA

## Abstract

**Background:**

Cerebral amyloid angiopathy (CAA), the accumulation of amyloid proteins in the cerebral vasculature, increases the risk of stroke and vascular cognitive impairment and dementia (VCID). Not only is there no treatment for CAA, but the condition is also highly comorbid with Alzheimer’s disease (AD), and its presence may serve as a contraindication to treating patients with anti‐amyloid therapies due to an increased risk of hemorrhage and edema. Therefore, it is crucial to identify novel treatments for individuals with CAA. Epidemiological studies suggest that certain antihypertensive medications, including those that target the renin‐angiotensin system (RAS), are associated with a decreased risk of dementia. This study assesses whether two FDA‐approved RAS‐targeting drugs: telmisartan [a moderately brain‐penetrant angiotensin receptor blocker (ARB)], and lisinopril [a brain‐penetrant angiotensin‐converting enzyme (ACE) inhibitor]; can be repurposed for the treatment of CAA.

**Methods:**

At either ∼3 months (early intervention) or ∼8 months (later intervention) of age, male and female Tg‐SwDI mice began treatment with either telmisartan (1 mg/kg/day) or lisinopril (15 mg/kg/day) dissolved in drinking water or received plain drinking water only. Age‐ and sex‐matched C57BL/6J mice receiving plain drinking water served as wild‐type controls. Following 4 months of treatment, mice underwent blood pressure measurement followed by behavioral testing prior to euthanasia.

**Results:**

Voluntary oral consumption delivered doses similar to the target dose for both drugs. At the doses used, telmisartan and lisinopril treatment did not significantly reduce blood pressure in Tg‐SwDI mice. Our findings thus far suggest that these drug treatments, particularly lisinopril, may mitigate cognitive‐behavioral deficits observed in Tg‐SwDI mice.

**Conclusions:**

Ongoing experiments are being completed to increase sample sizes and investigate the potential benefits of telmisartan and lisinopril to mitigate neuropathological and cognitive impairment in Tg‐SwDI mice. If findings support our hypothesis, this will demonstrate that these drugs could be repurposed to prevent and/or treat CAA, reducing the worldwide burden of stroke and dementia.

